# Antihypertensive and Immunomodulatory Effects of Defatted Corn Germ Hydrolysates: An *in vivo* Study

**DOI:** 10.3389/fnut.2021.679583

**Published:** 2021-05-24

**Authors:** Jiaqi Zhu, Jing Li, Yiting Guo, Janet Quaisie, Chen Hong, Haile Ma

**Affiliations:** ^1^School of Food and Biological Engineering, Jiangsu University, Zhenjiang, China; ^2^Institute of Food Physical Processing, Jiangsu University, Zhenjiang, China

**Keywords:** defatted corn germ hydrolysates, *in vivo* study, antihypertensive effect, tissue ACE activity, immunomodulatory effect

## Abstract

This study investigated the antihypertensive and immunomodulatory effects of defatted corn germ hydrolysates (DCGHs) *in vivo* and their potential regulatory mechanisms. The systolic blood pressure (SBP) of spontaneously hypertensive rats (SHRs) was significantly reduced (10.30%) by the long-term intragastric administration of DCGHs (high doses). Also, there was drastic inhibition of angiotensin-I-converting enzyme (ACE) activity in the lung, kidney, and heart tissues by 24.53, 22.28, and 12.93%, respectively. It could regulate the blood pressure by adjusting the balance between endothelium-derived vasoconstrictor factors and endothelium-derived relaxing factors. Meanwhile, DCGHs enhanced the phagocytosis of mononuclear macrophages, cellular immunity, and humoral immunity of ICR mice by increasing the phagocytic index of mononuclear macrophages (23.71%), ear swelling degree (44.82%), and antibody levels (52.32%). Moreover, it stimulated the release of immunoactive substances (e.g., lysozyme, interferon-γ, immunoglobulin G, and complement 3). Consequently, DCGHs could suitably be used in the formulation of novel functional foods with antihypertensive and immunomodulatory properties.

## Introduction

Defatted corn germ (DCG), a by-product of corn germ oil production, is often used as feed, resulting in a tremendous waste of nutritional value (1). The proteins in DCG are rich and balanced in most of the essential amino acids and mainly consist of soluble alkali proteins, albumins, and globulins, with a protein efficiency ratio similar to that of soy protein, and thus, DCG can be used as a high-quality protein source (2, 3). DCG can produce a significant quantity of active peptides *via* protease hydrolysis. Studies have shown that polypeptides have antihypertensive and immunomodulatory activities, which play an essential role in metabolic regulation and can serve as functional food ingredients, nutraceuticals, and pharmaceutical additives to improve human health and prevent diseases (4–6).

Hypertension is a chronic disease in which the blood pressure is higher than usual [systolic blood pressure (SBP) ≥140 mmHg, diastolic blood pressure ≥90 mmHg] (7). It has the characteristics of high incidence and gradual rejuvenation and can cause various diseases, such as cardiovascular disease, nephropathy, and myocardial infarction (8). The control of blood pressure in the body is dependent on the regulation of the renin-angiotensin system (RAS), and the angiotensin-converting enzyme (ACE) plays a vital role in the RAS (9). In RAS, renin acts on angiotensinogen to convert it into angiotensin I (Ang I), and under the action of ACE, Ang I is converted into angiotensin II (Ang II), causing an increase in blood pressure (10). At present, the drugs used to treat hypertension mainly include calcium channel antagonists, ACE inhibitors, angiotensin receptor blockers, and β-receptor blockers. However, these drugs have many side effects, such as skin rashes, disturbance of taste, dry cough, and allergic reactions (11, 12). Maes et al. (13) reported that milk protein-derived peptides could lower blood pressure *in vivo* by inhibiting the release of endothelin-1 (ET-1) from the endothelial cells of spontaneously hypertensive rats (SHRs). Alashi et al. (14) indicated that rapeseed peptides induced antihypertensive effects on SHRs. Therefore, bioactive substances derived from natural sources, which exert non-toxic side effects and hypotensive effects, have gradually become the focus of research.

Immunity protects organisms against harmful foreign bacteria and viruses (15). The immune system of the body is regulated by the interactions among macrophages, lymphocytes, and their immunoactive substances, which is manifested in the promotion of the growth of the immune organs, proliferation of immune cells, and release of immunoactive substances (16). Mao et al. reported that casein peptides obtained *via* alkaline protease hydrolysis could mediate immunoregulation by stimulating the release of ConA-induced lymphocyte proliferation and interleukin (IL)-2 in mice (5). Sun et al. reported that the polypeptides from *Pleurotus eryngii* mycelium could exert immunomodulatory effects by promoting the proliferation of macrophages and stimulating the release of tumor necrosis factor-α (TNF-α), IL-6, nitric oxide (NO), and H_2_O_2_ (17). Hence, the study of food-derived substances with immunomodulatory properties is gradually receiving widespread research attention.

Therefore, the purpose of this study was to evaluate the antihypertensive and immunomodulatory effects of DCGHs through animal experiments to study the more effective utilization of corn germ oil by-products. The antihypertensive effect of DCGHs *in vivo* was evaluated in terms of body weight (BW), blood pressure, tissue ACE activity, and serum biochemical indices of SHRs after acute oral and long-term intragastric administration. Meanwhile, the immunoregulatory effect of DCGHs was studied by testing the immune organ indices, phagocytic index of mononuclear macrophages, ear swelling degree, antibody levels, and serum immunoactive substance levels in ICR mice.

## Materials and Methods

### Materials

DCG was provided by Sanxing Corn Industry Co., Ltd. (Shandong, China). ACE with an activity of 0.4 U mL^−1^ was obtained from pig lungs using the method described by Wu et al. (18). Captopril, 4-(2-hydroxyethyl)-1-piperazineethanesulfonic acid, and N-[3-(2-Furyl) acryloyl]-Phe-Gly-Gly were purchased from Sigma (Shanghai, China). Indian ink was purchased from Solarbio Technology Co., Ltd. (Beijing, China). The kits for measuring the levels of renin, Ang II, ET-1, NO, prostacyclin (PGI_2_), TNF-α, glutathione-S transferase (GST), creatine kinase isoenzyme (CK-MB), 2,4-dinitrofluorobenzene (DNFB), lysozyme, interferon-γ (IFN-γ), immunoglobulins (IgA, IgG, and IgM), and complement 3 (C3) were purchased from Senbeijia Biological Technology Co., Ltd. (Nanjing, China). In addition, 2% sheep red blood cell (SRBC) suspension was purchased from Senbeijia Biological Technology Co., Ltd. All other chemicals used in this study were of analytical grade and purchased from Sinopharm (Shanghai, China).

### Preparation of DCGHs

**Figure 1:**
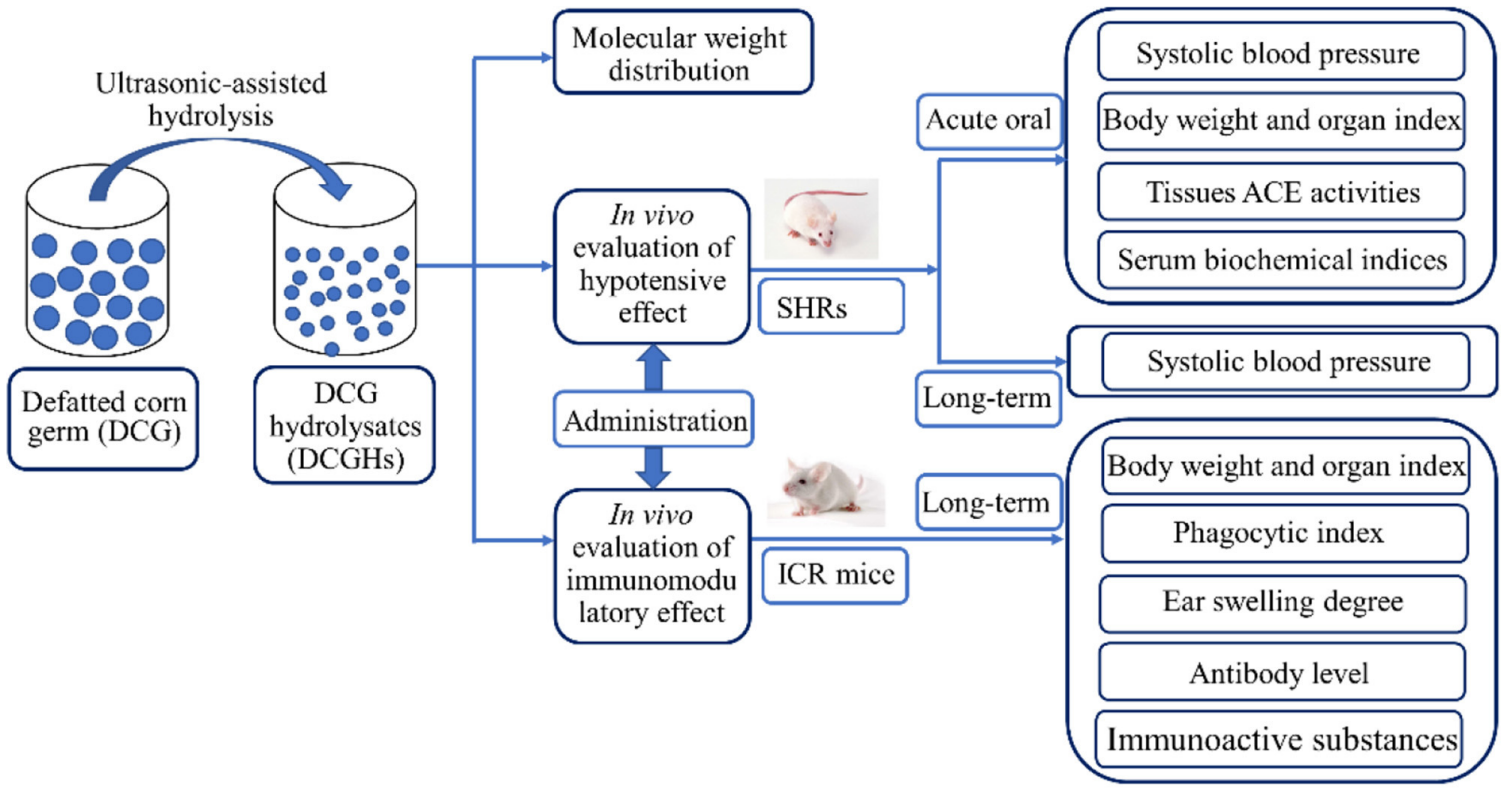
The flow chart of the preparation process and the *in vivo* study of DCGHs.

DCGHs was prepared from DCG using the ultrasonic-assisted enzymatic hydrolysis method, as described in a previous study (19). Briefly, energy-concentrated dual-frequency ultrasonic equipment was utilized in pretreating the substrate before enzymatic hydrolysis. Ultrasonic treatment was achieved by employing the optimal ultrasonic conditions from our preliminary study, that is, ultrasonic power density of 120 W/L, 15 min sonication time, and treatment temperature of 30°C. After the pretreatment, the substrate was subjected to enzymatic hydrolysis to obtain DCGHs under the hydrolysis conditions with a temperature of 50°C, a pH of 9.0, an enzyme dosage of 3,000 U/g, and reaction time of 30 min. From the above conditions, a total protein conversion degree of 63.34% was obtained. Figure 1 displays the preparation process and the *in vivo* study of DCGHs.

### Molecular Weight Distribution

The molecular weight distribution of DCGHs was determined using high-performance liquid size exclusion chromatography. The selected instrument and chromatographic column were Agilent 1100 liquid chromatography (Agilent, USA) and TSK-gel column (G2000, 300 × 7.8 mm, Tosoh, Tokyo, Japan), respectively. Bovine serum albumin (67,000 Da), cytochrome C (12,500 Da), bacitracin (1,450 Da), and L-tryptophan (204 Da) were used as standards.

### *In vivo* Blood Pressure-Lowering Experiment

#### Animals and Experimental Protocol

All the experiments were approved by the Institutional Animal Care and Use Committee of Jiangsu University and met the guidelines of the National Institutes of Health Guide for the Care and Use of Laboratory Animals. Thirty 13-week-old male-specific pathogen-free SHRs were purchased from Vital River Experimental Animal Technical Co. Ltd. (Beijing, China) (No. 11400700249349). They were raised in the barrier system of the Laboratory Animal Research Center of Jiangsu University. The conditions of the breeding room were temperature of 25 ± 2°C, humidity of 50 ± 5%, and daily light-dark cycle of 12 h. Before the administration test, the SHRs were subjected to a 1-week adaptation period. After a week of acclimatization, 30 SHRs were randomly divided into five groups (*n* = 6): control (administered a sterile saline solution), captopril (administered captopril at a dose of 30 mg/kg BW), low-dose (administered DCGHs at a dose of 500 mg/kg BW), middle-dose (administered DCGHs at a dose of 1,000 mg/kg BW), and high-dose (administered DCGHs at a dose of 1,500 mg/kg BW). At the beginning of the administration of these substances, the SBP of SHRs was measured and recorded every 3 from 0 to 12 h to perform the acute oral test. The SHRs were then intragastrically administered the aforementioned substances (depending on the group) daily for 5 weeks to perform a long-term administration test. The SBP of SHRs was measured using the tail-cuff method with an ALC-NIBP non-invasive blood pressure system once a week (Allcott Biotech Co. Ltd. Shanghai, China). Each measurement was repeated three times and the average was recorded. The same person performed all measurements at the same time of day and within the same environment to minimize blood pressure measurement errors.

#### Determination of Body Weight and Organ Index

In the long-term intragastric administration experiment, the BW of the SHRs was measured once a week. After 5 weeks, 1.5 mL of chloral hydrate solution (10%) was injected into the abdominal cavity of SHRs to induce anesthesia, following which the rats were euthanized and dissected. The celiac artery blood of the SHRs was collected using blood collection tubes not containing anticoagulants and stored at 25 ± 2°C for subsequent use. The heart, liver, spleen, lung, and kidney of SHRs were excised and weighed, and the organ index was calculated according to the following Equation (1):

(1) $$\begin{array}{l} \text{Organ index }\left(\%\right)\;=\;\frac{\text{Organ weight}}{\text{BW}}\;\times \;100\% \end{array}$$

#### Determination of Tissue ACE Activity

The extraction of ACE and determination of its activity in the lung, kidney, heart, and artery were performed using the method described by Guo et al. (20).

#### Biochemical Index Analysis of Serum

The celiac artery blood of the SHRs collected as described in section Determination of Body Weight and Organ Index was stored for 1 h to coagulate spontaneously and then centrifuged at 3,000 rpm for 10 min. The supernatant (serum) was aliquoted into different centrifuge tubes and stored at 4°C for further use. The levels of renin, Ang II, ET-1, NO, PGI_2_, TNF-α, GST, and CK-MB in the serum of the SHRs were determined using the experimental kits described in section Materials. The operation method referred to the kit instructions (20).

### *In vivo* Immunomodulation Experiment Arterial

#### Animals and Experimental Protocol

One hundred and sixty 6-week-old female-specific pathogen-free ICR mice were obtained from the Laboratory Animal Research Center of Jiangsu University (Jiangsu, China) (No. 201728221; No. 201726607). The breeding conditions of the ICR mice were the same as those of the SHRs and complied with the animal experiment guidelines described in section Animals and Experimental Protocol. The immunomodulatory effect of the ICR mice was evaluated using four tests: I (carbon clearance test), II (delayed-type hypersensitivity reaction (DTH) test), III (serum hemolysin test), and IV (serum immunoactive substance test). Each test (I, II, III, and IV) consisted of 40 ICR mice, which were randomly divided into four groups (*n* = 10): control (administered a sterile saline solution), low-dose (DCGHs administered at a dose of 100 mg/kg BW), middle-dose (DCGHs administered at a dose of 500 mg/kg BW), and high-dose groups (DCGHs administered at a dose of 1,000 mg/kg BW). These substances were administered intragastrically daily for 5 weeks. After the fifth week, the BW of ICR mice in the four tests were recorded and the body weight gain before and after the long-term administration experiment was calculated. Simultaneously, the spleen, thymus, heart, lung, liver, and kidney of ICR mice subjected to the test IV were excised and weighed to study the effect of DCGHs on the organ index of ICR mice. The organ index was calculated by Equation (1) in section Determination of Body Weight and Organ Index. Then, ICR mice subjected to tests I, II, III, and IV underwent carbon clearance, DTH, serum hemolysin, and serum immunoactive substance tests, respectively, to analyze the immunomodulatory effect of DCGHs on the body after long-term intragastric administration.

#### Determination of Macrophage Phagocytic Index, Ear Swelling Degree, and Antibody Levels

The macrophage phagocytic index, ear swelling degree, and antibody levels of ICR mice were measured using the carbon clearance (I), DTH (II), and serum hemolysin (III) tests, respectively.

The carbon clearance test (I) referred to the method described by Suzuki et al. (21) with some modifications. After long-term intragastric administration, the tail vein of ICR mice was injected with diluted Indian ink (100 mL/kg BW). Blood samples (20 μL) were collected from the retro-orbital venous plexus of ICR mice at the second and tenth minute after the injection and then immediately added to 2 mL of 0.1% Na_2_CO_3_ solution. The optical density (OD) at 600 nm was measured using a Multiskan spectrum microplate spectrophotometer (Infinite 200 PRO, Tecan Co., Ltd., Germany), and Na_2_CO_3_ solution was used as the control. The phagocytosis index was calculated using the following equation:

(2) $$\begin{array}{l} \text{Phagocytic index}=\;\frac{\text{BW}}{\text{Liver weight }+\text{ Spleen weight}}\\ \;\times \;\sqrt[{3}]{\frac{{\text{lgOD}}_{1}-{\text{lgOD}}_{2}}{{\text{t}}_{2}-{\text{t}}_{1}}} \end{array}$$

where OD_1_ and OD_2_ are the ODs at times t_1_ (second minute) and t_2_ (tenth minute), respectively.

The DTH test (II) was performed as described by Sakai et al. (22) with some modifications. The abdominal skin of each mouse was wiped with barium sulfide, shaved, and then 50 μL of 1% DNFB in acetone-seed oil (1:1) was evenly applied to the area to sensitize the skin of the mice. After 5 days, the right ears were challenged with 10 μL of 1% DNFB in acetone-seed oil (1:1), and the left ears were untreated. After 24 h, the ears with a diameter of 8 mm were removed using a hole punch and weighed. Ear swelling was expressed as the weight difference between the left and right ears.

The serum hemolysin test (III) was performed as described by Tort et al. (23). After the long-term administration experiment, the ICR mice in test III were intraperitoneally injected with 0.2 mL 2% SRBC. After 5 days, the retro-orbital venous plexus blood of ICR mice was collected in blood collection tubes not containing anticoagulants and stored at room temperature for 1 h for spontaneous blood coagulation. Next, the blood samples were centrifuged at 3,000 rpm for 10 min, and the serum was collected and diluted different multiples by the double dilution method. Then, 100 μL serum of different concentrations and 100 μL 0.5% (v/v) SRBC suspension were added to the micro hemagglutination test plate in turn. After mixing evenly, they were placed in a moist flat plate and incubated in an incubator at 37°C for 3 h. The degree of hemagglutination was observed and the antibody level was calculated by the following equation:

(3) $$\begin{array}{l} \text{Antibody level }=\;{\text{S}}_{1}\;+\;2{\text{S}}_{2}\;+\;3{\text{S}}_{3}\;+\ldots +\;{\text{nS}}_{\text{n}} \end{array}$$

where 1, 2, 3…, *n*, represent the index of double dilution, S represents the level of agglutination.

#### Determination of Serum Immunoactive Substances

The process of immune regulation is often accompanied by changes in the content of immunoactive substances. The ICR mice subjected to test IV underwent a serum immunoactive substances test, and the determination method referred to the article by Huang et al. (16). The serum of ICR mice was collected by the aforementioned method in the serum hemolysin test (III). After centrifugation, the serum was aliquoted into different centrifuge tubes and stored at 4°C for use. The levels of lysozyme, IFN-γ, IgA, IgG, IgM, and C3 in the serum of ICR mice were determined using the experimental kits described in section Materials. The operation method referred to the kit instructions (17).

### Statistical Analysis

The experimental data were expressed as the mean ± standard deviation (SD). The differences between groups were evaluated using the one-way analysis of variance (ANOVA). The statistical tests were analyzed using SPSS 19.0 (SPSS Inc., USA) at the significance levels of *p* < 0.05 and *p* < 0.01.

## Results and Discussion

### Molecular Weight Distribution

The molecular weight composition of peptides is an important index to evaluate the nutritional value of enzymatic hydrolysate, which is closely related to the absorption rate and physiological activity of enzymatic hydrolysate (24, 25). The molecular weight distributions of the DCGHs prepared using ultrasonic-assisted enzymatic hydrolysis are presented in Table 1. The fraction with a molecular weight of more than 2,000 Da accounted for the largest proportion (36.56%), followed by the range of 200–1,000 Da (35.97%), 1,000–2,000 Da (17.15%), and <200 Da (10.32%). Studies have shown that the polypeptide fractions with larger relative molecular weight prepared by hydrolysis could effectively utilize the protease in the gastrointestinal tract and have high biological activity after gastrointestinal digestion, even reaching or exceeding the functional activity level of traditional polypeptides (26). The result showed that DCGHs with molecular weight >2,000 and 200–1,000 Da accounted for more than 80%, which is similar to the results of the molecular weight distribution of walnut proteolysis products studied by Liu et al. (27), indicating that DCGHs may have good biological activity for application.

**Table 1:** Molecular weight distribution of defatted corn germ hydrolysates (DCGHs).

**Molecular weight distribution range (Da)**	**>2,000**	**1,000–2,000**	**200–1,000**	** <200**
Percentage (%)	36.56 ± 0.45	17.15 ± 0.37	35.97 ± 0.14	10.32 ± 0.31

### *In vivo* Blood Pressure-Lowering Experiment

#### BW and Organ Indices of the SHRs

The BW gain and organ indices are shown in Table 2. In the whole experiment, the BW gain of the SHRs in the tested groups was not significantly different from that of the control group (*p* > 0.05). In contrast, the BW gain of the SHRs in the captopril group was significantly lower than that in the control group (*p* < 0.01). Although captopril (an ACE inhibitor) has been prescribed for the treatment of cardiovascular disease, long-term use could cause some side effects such as vascular edema, rash, and dyspepsia (28). Hence, the weight of SHRs in the captopril group was significantly lower than that of the control group. Also, the organ indices of the SHRs in the tested groups and captopril group were not significantly different from those in the control group (*p* > 0.05), indicating that DCGHs and captopril did not affect the growth of organs in SHRs.

**Table 2:** Body weight and organ indices of SHRs.

**Group**	**Body weight gain**					**Organ indices**				
	**1st wk**	**2nd wk**	**3rd wk**	**4th wk**	**5th wk**	**Heart**	**Liver**	**Spleen**	**Lung**	**Kidney**
Control	2.12 ± 0.37	7.28 ± 0.68	10.68 ± 0.81	14.68 ± 1.32	19.01 ± 3.08	0.37 ± 0.01	2.48 ± 0.07	0.15 ± 0.01	0.34 ± 0.02	0.60 ± 0.02
Low	2.02 ± 0.42	7.40 ± 0.54	11.40 ± 0.77	13.95 ± 1.08	17.88 ± 2.52	0.35 ± 0.01	2.49 ± 0.07	0.14 ± 0.03	0.35 ± 0.03	0.59 ± 0.02
Middle	2.57 ± 0.35	6.98 ± 0.59	11.24 ± 0.90	14.80 ± 1.19	19.78 ± 1.97	0.36 ± 0.01	2.50 ± 0.08	0.16 ± 0.03	0.35 ± 0.10	0.59 ± 0.03
High	2.27 ± 0.22	7.18 ± 0.70	10.84 ± 0.83	15.37 ± 0.99	18.52 ± 1.26	0.40 ± 0.03	2.50 ± 0.09	0.16 ± 0.01	0.36 ± 0.05	0.61 ± 0.06
Cap	1.45 ± 0.18^*^	4.30 ± 0.34^**^	6.53 ± 0.62^**^	7.70 ± 0.93^**^	10.54 ± 2.30^**^	0.38 ± 0.03	2.48 ± 0.08	0.16 ± 0.01	0.36 ± 0.02	0.66 ± 0.05

*Control represents the control group (sterile saline solution); low, middle, and high represent the low-dose, middle-dose, and high-dose groups of defatted corn germ hydrolysates (DCGHs), respectively; Cap represents the captopril group*.

* *Significant differences (p < 0.05) compared to the control group*.

** *Significant differences (p < 0.01) compared to the control group*.

#### Blood Pressure of SHRs

**Figure 2:**
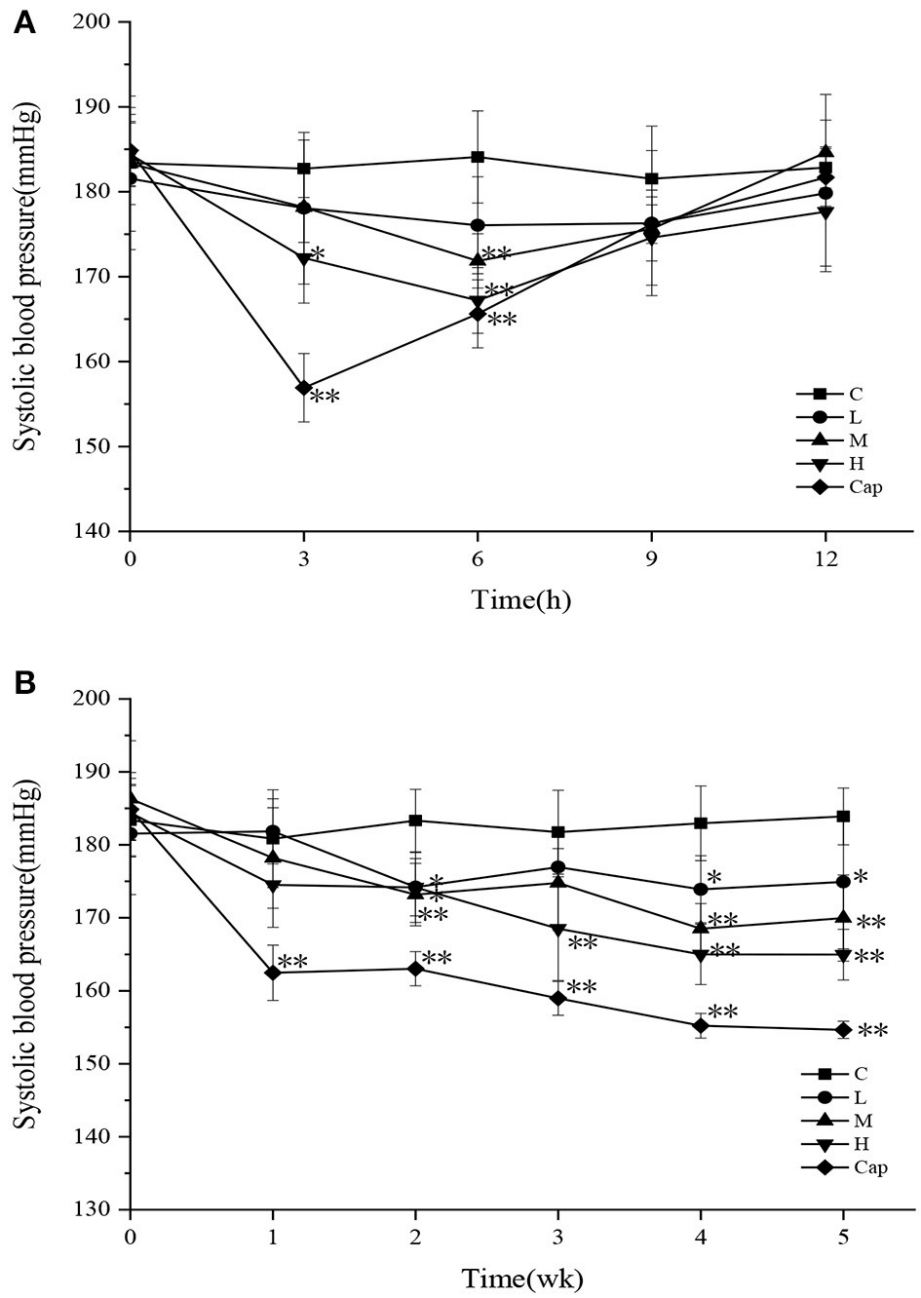
Effect of defatted corn germ hydrolysates (DCGHs) on systolic blood pressure (SBP) of spontaneously hypertensive rats (SHRs). Acute oral administration **(A)** and long-term intragastric administration **(B)**. C represents the control group (sterile saline solution); L, M, and H represent the low-dose, middle-dose, and high-dose groups of mice administered DCGHs, respectively; Cap represents the captopril group. *Significantly different (*p* < 0.05) and **Significantly different (*p* < 0.01) compared to C (sterile saline solution) at the same time point.

In the acute oral intragastric administration experiment, variations in SBP in different groups of SHRs are shown in Figure 2A. The SBP of the control group showed minimal change within 12 h. Compared with the control group, the SBP of the high-dose and captopril groups decreased significantly during the first 3 h (*p* < 0.05), especially in the captopril group (*p* < 0.01), which reached the maximum decrease of 25.81 mmHg. At 6 h, the SBP of the captopril group showed a significant reduction of 18.46 mmHg compared with the control group (*p* < 0.01). In the meantime, the SBP of the middle-dose and high-dose groups was significantly lower than that of the control group (*p* < 0.01), decreasing by 12.23 and 16.89 mmHg, respectively. After 9 h, the SBP of the tested groups and captopril group gradually returned to the initial level. Although the captopril group exerted a more rapid blood pressure-lowering effect than the tested groups initially, there was no difference between the tested groups and the captopril group after 6 h. Cui et al. (29) studied the antihypertensive effect of milk macromolecular peptides in the acute oral intragastric administration experiment. They found that the blood pressure of SHRs in the tested groups also reached the maximum decline at 6 h. The results initially showed that DCGHs had an antihypertensive effect *in vivo* in a short time, which was consistent with the result of milk macromolecular peptides.

The SBP of the control, tested, and captopril groups during the long-term intragastric administration test is shown in Figure 2B. The SBP of the captopril group was significantly lower than that of the control group from the first week to the fifth week and reached the maximum decrease of 29.25 mmHg at the fifth week (*p* < 0.01), revealing that the antihypertensive effect of captopril was rapid and long-lasting. All the tested groups presented a significant antihypertensive effect on SHRs from the second week (*p* < 0.05). The SBP of the high-dose group decreased more rapidly than that of the control group in the second week and maintained a downward trend until the fifth week, which attained the maximum decrease of 18.94 mmHg. In comparison with the control group, the SBP of the low-dose and middle-dose groups tended to be stable from the fourth week to the last week and decreased by 8.97 mmHg (*p* < 0.05) and 13.93 mmHg (*p* < 0.01), respectively, in the last week. The results showed that different doses of DCGHs also had a long-term and stable antihypertensive effect on SHRs, especially in the high-dose group. Wu et al. (30) found that different doses of ACE inhibitory peptides prepared from soy protein could significantly reduce the blood pressure of SHRs compared with the control group, which was consistent with our results. Therefore, DCGHs has good antihypertensive effect *in vivo*.

#### ACE Activity in the Tissues of SHRs

The ACE activity values are presented in Table 3. The ACE activity of the lung, kidney, heart, and aorta in the tested groups and captopril group decreased to different degrees than those in the control group. In comparison with the control group, ACE activity in the lung tissues of the middle-dose, high-dose, and captopril groups exhibited a significant reduction of 17.10, 24.53, and 30.83% (*p* < 0.01), while the low-dose group showed no significant changes (*p* > 0.05). Compared to the control group, ACE activity in the kidney and heart tissues of the high-dose group showed a significant reduction of 22.28% (*p* < 0.01) and 12.93% (*p* < 0.05), respectively, and the captopril group exhibited a marked decrease of 34.11% (*p* < 0.01) and 25.70% (*p* < 0.01), respectively, while the other tested groups showed minimal changes (*p* > 0.05). There were limited changes in ACE activity in the aorta among the control, tested, and captopril groups. Previous studies have shown that ACE exists widely in epithelial cells, neuroepithelial cells, and vascular endothelial cells in many organs (31). As shown in Table 3, the low-dose group showed limited effects on ACE activity in the tissues, the middle-dose group showed significantly reduced ACE activity in the lung tissues, and the high-dose and captopril groups showed significantly reduced ACE activity in the lung, kidney, and heart tissues. Moreover, DCGHs showed a dose-response relationship with ACE inhibitory activity. Therefore, DCGHs could exert an antihypertensive effect by inhibiting ACE activity in the lung, kidney, and heart tissues. Guo et al. (32) found that the corn germ peptides, prepared by an enzymatic membrane reactor with a new gradient diafiltration feeding working-mode (processing duration of 660 min), could significantly increase the tissue ACE activity of SHRs in a long-term intragastric administration. DCGHs can therefore be compared to corn germ peptides since they have similar ACE inhibitory activity in the body, and its preparation process (ultrasonic-assisted) makes full use of the protein resources of DCG, which dramatically shortens the enzymatic hydrolysis time and reduces energy consumption.

**Table 3:** ACE activity in the different tissues of SHRs.

**Group**	**Lung**	**Kidney**	**Heart**	**Aorta**
Control	946.93 ± 86.71	996.04 ± 66.71	739.81 ± 29.83	1436.04 ± 69.83
Low	910.48 ± 53.7	908.70 ± 83.70	728.70 ± 25.67	1392.00 ± 75.67
Middle	785.84 ± 68.70^**^	911.67 ± 38.73	711.67 ± 72.02	1347.01 ± 72.00
High	714.64 ± 41.67^**^	774.08 ± 61.67^**^	644.08 ± 47.01^*^	1381.67 ± 67.00
Cap	654.93 ± 34.08^**^	656.20 ± 84.08^**^	549.67 ± 61.67^**^	1343.60 ± 31.67

*Control represents the control group (sterile saline solution); low, middle, and high represent the low-dose, middle-dose, and high-dose groups of defatted corn germ hydrolysates (DCGHs), respectively; Cap represents the captopril group*.

* *Significant differences (p <0.05) compared to the control group*.

** *Significant differences (p <0.01) compared to the control group*.

#### Serum Biochemical Indices of SHRs

The endothelium-derived vasoconstrictor factors (EDCFs) and endothelium-derived relaxing factors (EDRFs) secreted by vascular endothelial cells can regulate blood pressure and endothelial function (20). To further explore the mechanism underlying the antihypertensive effects of DCGHs *in vivo*, the serum biochemical indicators of SHRs were analyzed. As shown in Table 4, compared with the control group, the content of renin in the middle-dose and high-dose groups showed a marked decrease of 14.01 and 14.62% (*p* < 0.05), while the content of renin in the captopril group significantly increased by 12.57% (*p* < 0.05). In addition to the low-dose group, the content of Ang II in the middle-dose, high-dose, and captopril groups was significantly different from that in the control group (*p* < 0.01), representing a decrease of 10.60, 10.80, and 13.30%, respectively. Renin (an EDCF) initiates the RAS chain reaction and causes an increase in blood pressure. The expression of RAS promotes the production of Ang II (an EDCF), which regulates vasoconstriction and stimulates the vascular endothelial cells to secrete ET-1 (an EDCF) (33). The results showed that DCGHs could regulate blood pressure by promoting the production of renin and Ang II, while captopril had a rapid antihypertensive effect, resulting in a fast decrease in Ang II levels. This led to an increase in renin content to maintain the balance of the RAS. As shown in Table 4, the content of ET-1 (an EDCF) in the high-dose and captopril groups decreased significantly by 23.62% (*p* < 0.01) and 28.17% (*p* < 0.01), respectively, compared with the control group. The content of NO (an EDRF) in the tested and captopril groups increased significantly compared with the control group (*p* < 0.05); particularly, in the high-dose group, NO content increased by 36.53% (*p* < 0.01). ET-1 (an EDCF), a subtype of endothelin, has the effect of regulating vasoconstriction (13). NO (an EDRF) has the effect of protecting vascular smooth muscle tissue, regulating angiogenesis, and preventing harmful blood vessel contraction (34). The results indicated that DCGHs could exert a hypotensive effect by inhibiting the release of ET-1 and promoting the release of NO. PGI_2_ (an EDRF) can relax smooth muscle cells of different blood vessel beds to regulate systemic vascular tone (35). The results in Table 4 show that PGI_2_ content in the tested groups showed less change compared with the control group (*p* > 0.05), while that of the captopril group increased significantly by 21.58% compared with the control group (*p* < 0.01). Therefore, DCGHs had little effect on blood pressure regulation through PGI_2_. The increase in TNF-α (an EDCF) content in the serum can cause hypertrophy and proliferation of vascular smooth muscle cells, leading to an increase in blood pressure (36). As described in Table 4, the content of TNF-α in the tested and captopril groups was significantly higher than that in the control group (*p* < 0.05), indicating that DCGHs can regulate blood pressure through TNF-α. GST has antioxidative and detoxifying effects, and its concentration changes in serum reflect the degree of damage to the liver (37). The concentration of CK-MB in the serum can increase to a certain extent due to myocardial injury or necrosis (38). As observed in Table 4, in comparison with the control group, the content of GST and CK-MB in the tested groups showed less change (*p* > 0.05), while the content of GST and CK-MB in the captopril group increased by 15.81% (*p* < 0.01) and 6.63% (*p* < 0.05), respectively. Captopril possibly caused damage to the myocardium and liver of SHRs, and DCGHs were safe for the body of SHRs. Thus, the assessment of the serum biochemical indicators showed that the mechanism underlying the antihypertensive effects of DCGHs involved the regulation of the balance between EDCFs and EDRFs in the serum (39).

**Table 4:** Serum biochemical indices of SHRs after long-term intragastric administration.

**Group**	**Renin (ng/L)**	**Ang II (ng/L)**	**PGI_**2**_ (ng/L)**	**ET-1 (ng/L)**	**TNF-α (ng/L)**	**NO (μmol/L)**	**GST (ng/mL)**	**CK-MB (ng/mL)**
Control	74.60 ± 4.10	174.17 ± 7.07	109.29 ± 10.58	81.25 ± 8.20	8.28 ± 0.28	20.23 ± 1.84	22.07 ± 1.51	133.00 ± 4.43
Low	75.42 ± 7.72	167.41 ± 5.51	106.27 ± 11.33	80.22 ± 6.95	7.61 ± 0.12^*^	25.03 ± 2.84	23.68 ± 1.00	135.81 ± 2.43
Middle	64.15 ± 3.08^*^	155.70 ± 5.16^**^	108.68 ± 6.68	78.11 ± 4.96	7.63 ± 0.51^*^	25.58 ± 3.70^*^	22.90 ± 0.74	130.72 ± 2.70
High	63.69 ± 3.10^*^	155.36 ± 7.46^**^	113.68 ± 8.05	62.06 ± 9.17^**^	7.57 ± 0.18^*^	27.62 ± 2.28^**^	23.43 ± 1.07	130.64 ± 2.70
Cap	83.98 ± 4.47^*^	151.01 ± 7.91^**^	132.88 ± 8.05^**^	58.36 ± 5.80^**^	7.41 ± 0.39^**^	22.19 ± 2.55	25.56 ± 0.90^**^	141.82 ± 8.93^*^

*Control represents the control group (sterile saline solution); low, middle, and high represent the low-dose, middle-dose, and high-dose groups of defatted corn germ hydrolysates (DCGHs), respectively; Cap represents the captopril group*.

* *Significant differences (p <0.05) compared to the control group*.

** *Significant differences (p <0.01) compared to the control group*.

### *In vivo* Immunomodulation Experiment

#### BW and Organ Indices of ICR Mice

The results of BW gain and organ indices of ICR mice in the long-term intragastric administration experiment are shown in Table 5. For the tests I (carbon clearance test), II (DTH test), III (serum hemolysin test), and IV (serum immunoactive substance test), the BW gain of ICR mice in the tested groups (the low-dose, middle-dose, and high-dose groups) did not change significantly compared with the control group (*p* > 0.05). It demonstrated that the treatment of DCGHs had no side effect on the growth rate of BW. Similarly, for test IV (serum immunoactive substance test), the weights of the spleen, thymus, heart, lung, liver, and kidney of ICR mice between the control group and tested groups were not significantly different (*p* > 0.05), which confirmed the non-toxic side effects and safety use of DCGHs. Therefore, DCGHs can be preliminarily used as a food-borne substance that is harmless to the body.

**Table 5:** Body weight and organ indices of ICR mice.

**Group**	**Body weight gain**				**Organ indices**					
	**I**	**II**	**III**	**IV**	**Spleen**	**Thymus**	**Heart**	**Lung**	**Liver**	**Kidney**
Control	7.57 ± 2.01	7.85 ± 1.68	7.34 ± 1.21	7.23 ± 1.74	0.33 ± 0.06	0.29 ± 0.05	0.49 ± 0.03	0.62 ± 0.06	4.06 ± 0.25	1.07 ± 0.11
Low	7.35 ± 1.45	7.47 ± 1.90	7.72 ± 1.54	7.47 ± 2.10	0.34 ± 0.08	0.29 ± 0.04	0.43 ± 0.06	0.57 ± 0.01	4.05 ± 0.27	1.08 ± 0.12
Middle	7.75 ± 2.16	7.67 ± 1.74	7.53 ± 1.22	7.65 ± 1.41	0.34 ± 0.09	0.30 ± 0.05	0.45 ± 0.10	0.53 ± 0.07	4.01 ± 0.32	1.06 ± 0.05
High	7.13 ± 1.52	7.22 ± 1.55	7.24 ± 2.08	6.98 ± 1.33	0.36 ± 0.06	0.31 ± 0.03	0.45 ± 0.03	0.54 ± 0.04	4.03 ± 0.10	1.09 ± 0.09

*I, II, III, and IV represent carbon clearance test, delayed-type hypersensitivity reaction (DTH) test, serum hemolysin test, and serum immunoactive substance test, respectively. Control represents the control group (sterile saline solution); low, middle, and high represent the low-dose, middle-dose, and high-dose groups of ICR mice administered defatted corn germ hydrolysates (DCGHs), respectively*.

#### Macrophage Phagocytic Index, Ear Swelling Degree, and Antibody Levels of ICR Mice

The results of the carbon clearance, DTH, and serum hemolysin tests are shown in Table 6, which reflect the phagocytic function of mononuclear macrophages, cellular immune function, and humoral immune function, respectively (40). The phagocytic index of mononuclear macrophages in the middle-dose and high-dose groups was significantly higher than that in the control group, increasing by 17.18% (*p* < 0.01) and 23.71% (*p* < 0.01), respectively. The low-dose group showed less difference compared with the control group (*p* > 0.05). The phagocytic index reflects the phagocytic ability of mononuclear macrophages. The higher the phagocytic index, the stronger the phagocytic capacity. The results showed that DCGHs could improve the phagocytic capacity of mononuclear macrophages in ICR mice. DTH reflects the level of cellular immune function, and the magnitude of it is expressed by ear swelling degree. As shown in Table 6, the ear swelling degree of ICR mice in the low-dose group was not significantly different from that of the control group, while the ear swelling degree of ICR mice in the middle-dose and high-dose groups was 21.95% (*p* < 0.05) and 44.82% (*p* < 0.01) higher than that of the mice in the control group, indicating that DCGHs could improve the cellular immune function of ICR mice. Antibody levels reflect the humoral immune function of the body. In comparison with the control group, antibody levels in the high-dose group increased significantly by 52.32% (*p* < 0.01), while the levels in the other tested groups showed minimal changes (*p* > 0.05). The serum hemolysin test results revealed that DCGHs could significantly enhance the humoral immune function of ICR mice. Huang et al. (41) found that detoxified rapeseed peptides had a significant effect on the phagocytic ability and ear swelling degree of ICR mice, which presented a good regulatory effect on immunity. Therefore, DCGHs also have a good immunomodulatory effect *in vivo* compared with the detoxified rapeseed peptides.

**Table 6:** Macrophage phagocytic index, ear swelling degree, and antibody levels of ICR mice.

**Group**	**Phagocytic index**	**Ear swelling mg**	**Antibody levels**
Control	8.73 ± 0.86	15.17 ± 4.65	35.59 ± 3.11
Low	9.00 ± 0.36	16.80 ± 2.69	37.97 ± 5.20
Middle	10.23 ± 0.41^**^	18.50 ± 3.08^*^	40.41 ± 4.67
High	10.80 ± 0.96^**^	21.97 ± 4.50^**^	54.21 ± 5.63^**^

*Control represents the control group (sterile saline solution); low, middle, and high represent the low-dose, middle-dose, and high-dose groups of ICR mice administered defatted corn germ hydrolysates (DCGHs), respectively*.

* *Significant differences (p < 0.05) compared to the control group*.

** *Significant differences (p < 0.01) compared to the control group*.

#### Immunoactive Substances in the Serum of ICR Mice

To further explore the immunomodulatory effects of DCGHs *in vivo*, the immunoactive substances in the serum of ICR mice were determined. As shown in Table 7, lysozyme content in the tested groups was significantly higher than that in the control group (*p* < 0.05). Particularly, in the high-dose group, lysozyme content increased by 34.97% (*p* < 0.01). Lysozyme can kill pathogenic bacteria and alleviate inflammation, reflecting the degree of non-specific immune regulation (42). Therefore, DCGHs promoted the release of lysozyme in ICR mice to regulate immune function. IFN-γ, a cytokine, can activate immune cells, improve the ability to kill pathogens and tumor cells, and promote phagocytosis of macrophages (43, 44). Table 7 shows that the content of IFN-γ in the high-dose group increased by 18.53% (*p* < 0.05) compared with the control group, but there was no significant change in the other tested groups (*p* > 0.05). Studies have shown that low levels of IFN-γ can cause immune system dysfunction in mice, resulting in a decline in immunity (45). Therefore, the results showed that DCGHs could stimulate the release of IFN-γ to enhance the immune function of the body. Immunoglobulin is a soluble globulin with humoral immune function. As shown in Table 7, IgA and IgM content in the tested groups was not significantly different from that in the control group (*p* > 0.05), while the content of IgG in the middle-dose and high-dose groups was significantly higher than that in the control group (*p* < 0.01), showing an increase of 38.42 and 21.72%, respectively. IgG levels were the highest in the serum, and this immunoglobulin has various immunomodulatory activities, such as antibacterial, antiviral, and antitoxin effects. It can also promote the connection between tumor cells and effector cells, causing cytotoxicity to kill tumor cells and other target cells (46). The results showed that DCGHs could act as immunomodulators by increasing the IgG content in the serum. C3, the most abundant protein in the complement system, can be cleaved into biologically active fragments after activation, which assists phagocytes and antibodies in killing pathogenic bacteria and strengthening immune function (47). As indicated in Table 7, C3 content in the middle-dose and high-dose groups was significantly higher than that in the control group (*p* < 0.01), increasing by 6.13 and 6.68%, respectively. The results showed that DCGHs could increase C3 levels in the serum of ICR mice to exert immunoregulatory effects. Ratajczak et al. (48) studied the good immune regulation function of corn oil *in vivo*. Lee et al. (49) found that corn byproduct poly-saccharides isolated from corn steep liquor could exert an immunomodulatory effect by stimulating the release of Immunoactive substances. Compared with the immunomodulatory effects of corn oil and corn byproduct poly-saccharides, DCGHs also have a good immune regulation effect, which provides a new guide for the comprehensive development and utilization of corn by-products.

**Table 7:** Serum immunoactive substances of ICR mice.

**Group**	**Lysozyme (μg/L)**	**IFN-γ (ng/L)**	**IgA (μg/mL)**	**IgG (μg/mL)**	**IgM (ng/mL)**	**C3 (μg/mL)**
Control	20.19 ± 2.23	912.83 ± 91.51	3.31 ± 0.34	9.76 ± 0.78	131.21 ± 11.27	455.16 ± 6.31
Low	24.22 ± 2.84^*^	959.58 ± 140.58	3.55 ± 0.38	10.94 ± 1.11	136.06 ± 8.71	463.19 ± 7.35
Middle	25.34 ± 3.56^*^	939.75 ± 156.81	3.63 ± 0.46	11.88 ± 1.54^*^	134.29 ± 18.19	483.07 ± 8.35^**^
High	27.25 ± 2.78^**^	1082.00 ± 111.21^*^	3.54 ± 0.43	13.51 ± 1.34^**^	137.81 ± 11.85	485.57 ± 11.68^**^

*Control represents the control group (sterile saline solution); Low, Middle, and High represent the low-dose, middle-dose, and high-dose groups of ICR mice administered defatted corn germ hydrolysates (DCGHs), respectively*.

* *Significant differences (p <0.05) compared to the control group*.

** *Significant differences (p <0.01) compared to the control group*.

## Conclusions

In this study, we investigated the antihypertensive and immunomodulatory effects of DCGHs *in vivo*. Analysis of tissue ACE activity demonstrated that DCGHs could exert an antihypertensive effect mainly by inhibiting ACE activity in the kidney, lung, and heart tissues. Moreover, the serum biochemical factor test results indicated that the DCGHs could adjust the balance between EDCFs (e.g., renin, Ang II, TNF-α, and ET-1) and EDRFs (e.g., NO) to regulate blood pressure. Furthermore, the results of analyses of the immunomodulatory effects showed that DCGHs could enhance the phagocytosis of mononuclear macrophages, cellular immunity, and humoral immunity by increasing the phagocytic index of mononuclear macrophages, ear swelling degree, and antibody levels in ICR mice, in addition to stimulating the release of immunoactive substances (e.g., lysozyme, IFN-γ, IgG, and C3). The results herein, may add to existing knowledge on the bioactive properties of DCGHs and guide the development and utilization of cereal-based by-products.

## Data Availability Statement

The original contributions presented in the study are included in the article/supplementary material, further inquiries can be directed to the corresponding author/s.

## Ethics Statement

The animal study was reviewed and approved by Institutional Animal Care and Use Committee of the Jiangsu University.

## Author Contributions

JZ: conceptualization, methodology, data curation, and writing-original draft. JL: investigation, software, and formal analysis. YG: writing-review and editing and formal analysis. CH: investigation and data curation. JQ: visualization and writing-review and editing. HM: project administration, funding acquisition, supervision, and resources. All authors contributed to the article and approved the submitted version.

## Conflict of Interest

The authors declare that the research was conducted in the absence of any commercial or financial relationships that could be construed as a potential conflict of interest.
